# RURAL–URBAN RESIDENCE, EDUCATION, AND SUBJECTIVE COGNITIVE DECLINE AMONG MIDDLE AGED AND OLDER ADULTS IN THE US

**DOI:** 10.1093/geroni/igae098.0420

**Published:** 2024-12-31

**Authors:** Roger Wong, Amer Mansour

**Affiliations:** State University of New York Upstate Medical University, New York, United States; State University of New York Upstate Medical University, Syracuse, New York, United States

## Abstract

Subjective cognitive decline (SCD) is defined as an individual’s self-reported experience of increased confusion or memory loss, and it is a known risk factor for dementia. There is no existing research on SCD disparities by rural-urban residence, and how education may play a role in this relationship. To address this gap, the 2022 Behavioral Risk Factor Surveillance System data was analyzed to elucidate the interplay between rurality, education, and SCD. The SCD measure was only collected from respondents aged 45 years and older, resulting in a sample size of 63,948 U.S. adults. SCD was significantly more common among rural (12.0%) compared to urban (10.7%) residents [X2(1)=15.5, p<.001]. In a multiple logistic regression model, rural residents had a 16% significantly higher odds of SCD compared to urban residents after adjusting for sociodemographic and health covariates (adjusted odds ratio [aOR]=1.16, 95% CI=1.01-1.33, p<.05). There was a negative relationship between education level and SCD, but insignificant. For example, a college degree was associated with 12.4% lower odds of SCD compared to those with less than high school education (aOR=0.88, 95% CI=0.70-1.09, p=.24). Education did not significantly moderate the association between rurality and SCD. Our results suggest rural residents have higher odds of experiencing SCD, but no level of education may buffer this relationship despite extensive research on the benefits of education on cognitive health. Future research is needed to examine the mechanisms contributing to higher levels of SCD among rural residents, and effective interventions that may be used to mitigate further cognitive decline.

